# Total portal vein replacement with peritoneal interposition graft during Whipple’s procedure for extrahepatic cholangiocarcinoma: a technical report

**DOI:** 10.1186/s12957-023-02995-x

**Published:** 2023-03-29

**Authors:** Rebecca Marino, Antonella Tudisco, Francesca Ratti, Federica Pedica, Luca Aldrighetti

**Affiliations:** 1 Hepatobiliary Surgery Division, IRCCS San Raffaele Hospital, 20132 Milan, Italy; 2 Faculty of Medicine, University Vita-Salute San Raffaele, 20132 Milan, Italy

**Keywords:** Distal cholangiocarcinoma, Pancreaticoduodenectomy, Graft, Parietal peritoneum, Hepatic surgery, Vascular reconstruction

## Abstract

**Background:**

Aggressive surgical resection in locally advanced hepatopancreatobiliary (HPB) malignancies is frequently advocated as the only potentially curative treatment. In recent years, advances in chemotherapy regimens and surgical techniques have led to improved oncologic outcomes and overall survival, by increasing the rates of radical (R0) resections. Vascular resections are increasingly reported to further increase disease clearance rates. Within this perspective, the issue of vascular reconstruction has raised growing interest, drawing particular attention to vascular substitutes and surgical techniques for reconstruction.

**Case presentation:**

A case of extrahepatic cholangiocarcinoma with high clinical suspicion of vascular infiltration of the portal trunk at preoperative assessment is reported. An autologous interposition graft, harvested from diaphragmatic peritoneum, was chosen as a vascular substitute leading to successful portal trunk reconstruction and overcoming all possible drawbacks associated with cadaveric and artificial grafts reconstructions.

**Conclusion:**

This solution was strategic to ensure complete oncologic clearance averting the risk of positive margins (R1) at final pathology.

## Introduction

With the rationale of ensuring radical tumor excision, vascular resections are increasingly common due to extensive infiltration of hilar structures, despite technical complexity [1]. Based on this background, growing interest in vascular reconstruction techniques has emerged. When approaching a locally advanced HPB malignancy, forecasting the need for vascular resection is often difficult. The presence and the entity of vascular involvement are not always accurately defined in preoperative imaging. Therefore, all possible intraoperative findings, without a specific contraindication to resection, such as unexpected intraoperative vascular invasion, need to be adequately tackled. If vascular infiltration is evident at preoperative imaging, the appropriate vascular substitute can be chosen beforehand. Among available vessel substitutes, autologous veins, cryopreserved vessels, synthetic polytetrafluorethylene (PTFE) graft, and autologous parietal peritoneum grafts (APG) are the most used [2–5].

APG was first described in the mid-twentieth century in preclinical studies [6]. It was then described as a viable vascular substitute for inferior vena cava (IVC) reconstruction and just recently it was described as a feasible alternative in pancreatic resections requiring mesentericoportal axis reconstruction [7, 8].

A case of extrahepatic cholangiocarcinoma, originating from the common hepatic duct, with high suspicion of portal trunk’s tumor infiltration is presented. The aim is to provide a technical report regarding the use of an APG as a vascular substitute exploiting its advantages as an effective and easily available reconstruction’s method.

## Case report

A 67-year-old woman, referred from a peripheral secondary care center, with a diagnosis of cholangiocarcinoma originating from the common hepatic duct was admitted to the Hepato-Biliary Surgery Division of San Raffaele Hospital, Milan. At admission, laboratory values were within normal ranges except for hyperbilirubinemia (total bilirubin levels: 15.6 mg/dl; normal range < 0.25 mg/dl) and cholestatic liver enzymes abnormalities (alkaline phosphatase levels: 450 U/L, normal range 33–141 U/L; gamma-glutamyl transferase levels: 160 U/L, normal range 6–40 U/L).

**Fig. 1:**
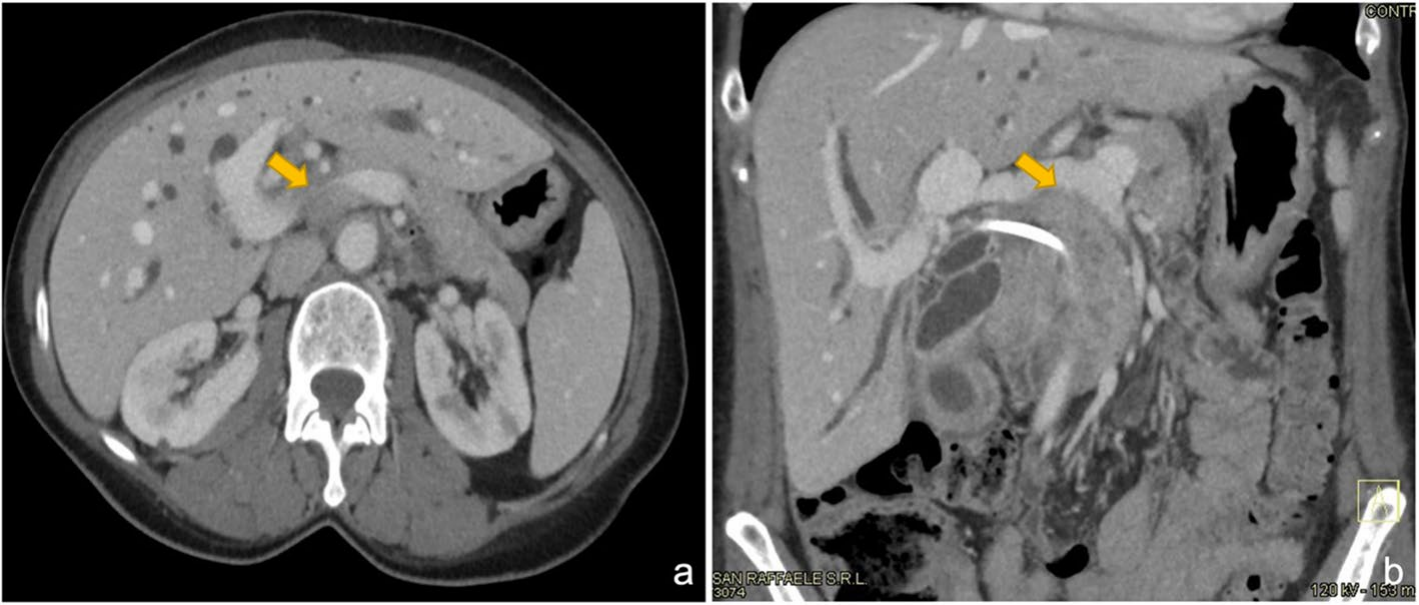
Axial (**a**) and coronal (**b**) preoperative CT sections showing narrowing of the portal trunk (yellow arrow) above spleno-mesenteric confluence

Preoperative CT (Fig. 1a, b) scan showed a mass arising from the middle third of the common hepatic duct. The cranio-caudal extension of the lesion caused a marked narrowing of portal vein’s caliber which was suspect for tumor encasement. Diffusion-weighted magnetic resonance confirmed CT scan findings and did not reveal further hepatic lesions.

Preoperative management included endoscopic retrograde cholangio-pancreatography (ERCP) plastic stenting resulting in effective biliary decompression and jaundice resolution. The preoperative management of this patient, including preoperative imaging and ERCP, was entirely planned and executed before referral to our center.

Upon referral, the case was discussed at our weekly multidisciplinary meeting specifically dedicated to biliary tumors and including surgeons, oncologists, radiologists, radiotherapists, and pathologists to define the treatment strategy within a patient-tailored management. Common bile duct (CBD) resection was planned, taking into consideration the possibility to extend the resection longitudinally in case of biliary involvement at intraoperative frozen section (i.e., to perform liver resection or pancreatic resection) and to perform a portal resection in case of radial vascular involvement.

Considering tumor’s location and vascular encasement, extemporaneous histological examination, of both proximal and distal margins, represented the turning point in guiding the resection’s extent. In case of intraoperative evidence of cranial extension, en bloc resection of common bile duct, biliary confluence, gallbladder, and liver segments 4b-5, possibly leading to a former major hepatectomy (e.g., in the presence of further cranial involvement), was planned, whereas, in case of intraoperative evidence of caudal extension, a Whipple procedure was planned.

This study was approved by the institutional review board. The need for written informed consent was waived.

### Surgical technique

A right hypochondrium square incision was performed to open the abdominal cavity. At careful surgical exploration, the gallbladder appeared overdistended due to neoplastic tissue embedding the common hepatic duct and infiltrating both the infundibulum and the superior duodenal flexure. Intraoperative liver ultrasound confirmed portal trunk narrowing above the spleno-mesenteric confluence. Station 8 and 12 lymphadenectomy was performed and full access to the hepatic arterial axis was obtained. The common hepatic duct was followed until the confluence; it was then dissected at his root and sectioned below the confluence. Cross section of both ducts’ stumps was obtained, and intraoperative histological examination was performed to assess bile ducts margins (negative margin status). After duodenal kocherization and exposure of the aortocaval area, the gastroduodenal artery was identified and sectioned. The lesser sac was entered, and left gastroepiploic vessels were isolated and sectioned along with the gastric body. The inferior pancreatic border was detached from the transverse mesocolon until full exposure of the superior mesenteric vein (SMV) was obtained; subsequently, pancreatic transection was performed. During careful detachment of the pancreatic head from the portal plane, the anterior wall resulted infiltrated by neoplastic tissue. Anterior mesenteric artery was identified and pancreaticoduodenectomy en bloc with gallbladder and segment 4 resection was completed. Due to the longitudinal extent of portal trunk invasion not allowing an end-to-end vessel reconstruction, total portal vein replacement with parietal peritoneum graft interposition seemed the best viable option. This strategy ensured both safe portal reconstruction and complete tumor excision with negative margins at final pathology. Whipple’s procedure ended with pancreatico- and gastro-jejunostomy. Biliary tract reconstruction was performed through a double termino-lateral hepaticojejunostomy Roux-en-Y anastomosis with PDS 4/0.

### Parietal peritoneum graft procurement

**Fig. 2:**
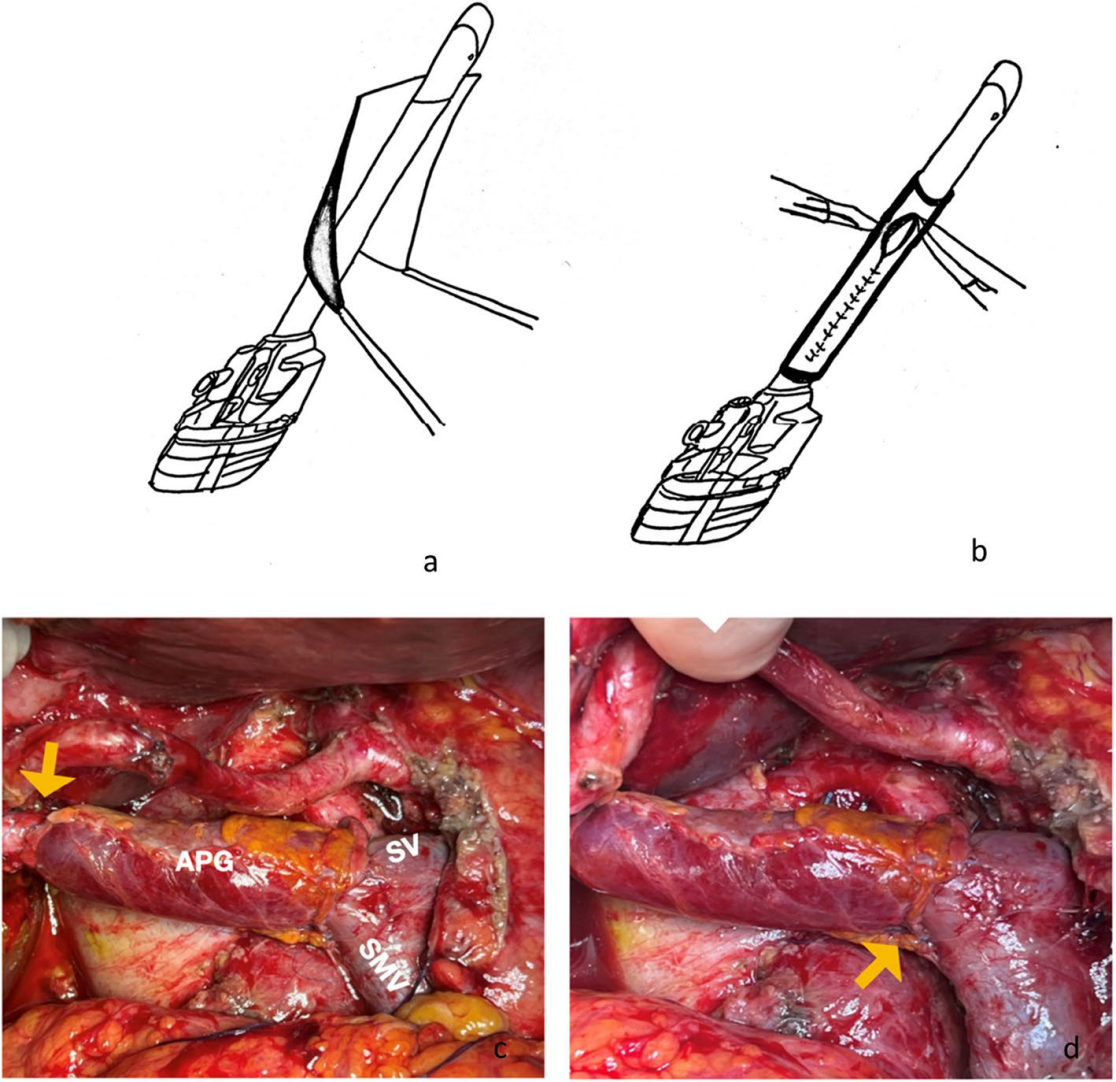
**a**,** b** Autologous peritoneal interposition graft preparation. A 4 × 3 cm section of diaphragmatic parietal peritoneum was harvested from the right subcostal area. The section obtained was tubularized, using a 10-mm trocar as support model (**a**) by a running 6/0 prolene suture (**b**). **c**,** d** Operating field after successful portal trunk reconstruction with autologous peritoneal interposition graft (APG). The graft was anastomosed (yellow arrows), under proximal and distal clamping, right above the spleno-mesenteric confluence with two end-to-end prolene 6/0 sutures. SV, splenic vein; SMV, superior mesenteric vein

A 4 × 3 cm section of diaphragmatic parietal peritoneum was harvested from the right subcostal area. The section obtain was tubularized, using a 10-mm trocar as support model, by a running 6/0 prolene suture. Before portal clamping, intravenous heparin (3000 units) was administered. The portal vein was entirely reconstructed with the interposition tube graft obtained and cut to length (Fig. 2a, b).

Ultimately, an end-to-end graft to portal vein anastomoses was completed with 6/0 prolene suture (Fig. 2c, d).

Adequate portal flow and patency were proven using intraoperative Doppler ultrasound.

### Postoperative assessment

**Fig. 3:**
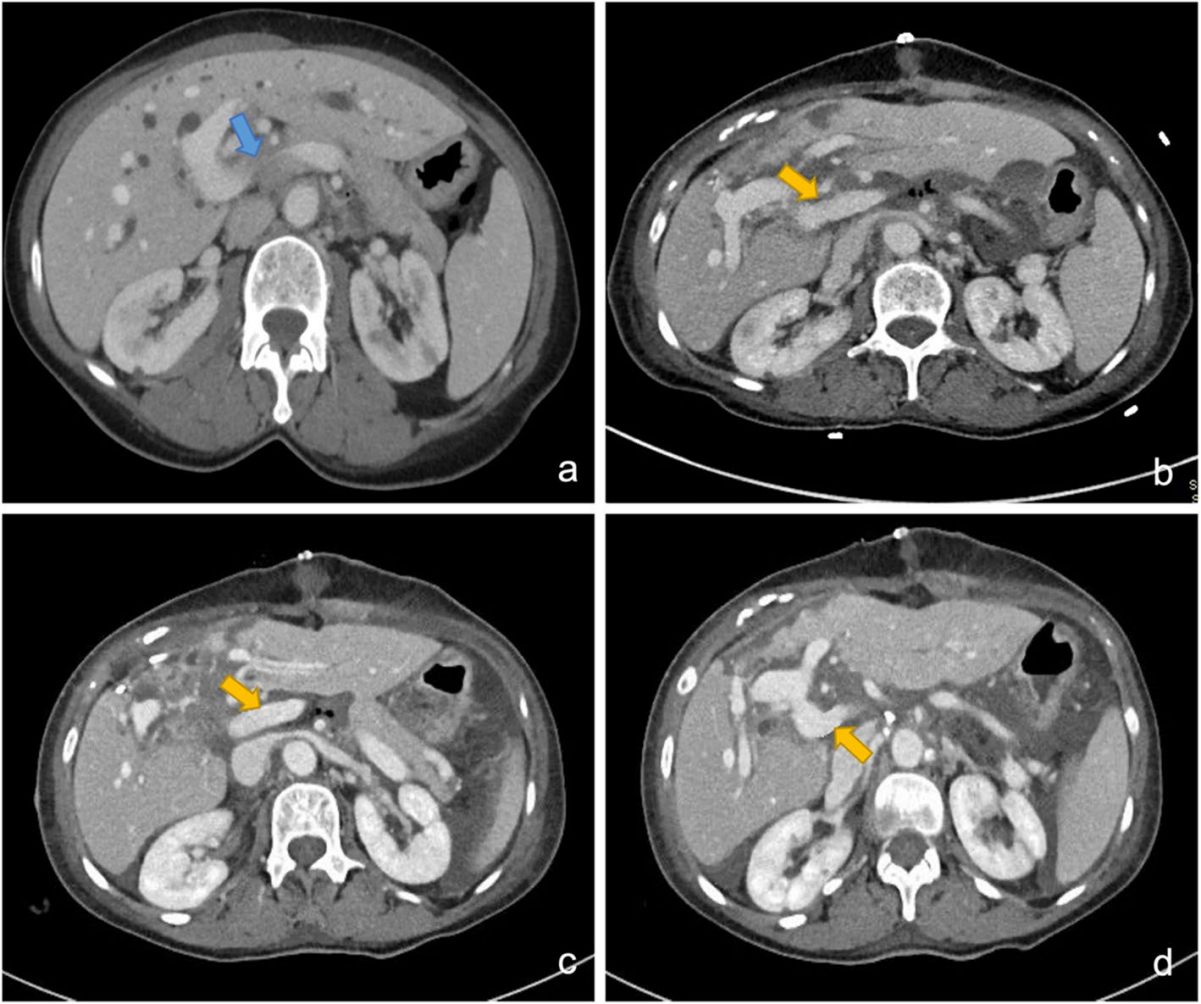
**a**, **b**, **c**, **d** Axial (**a**) preoperative CT section showing narrowing of portal trunk (blue arrow) above spleno-mesenteric confluence due to extensive tumor’s infiltration. Postoperative CT scans (**b**–**d**) demonstrating surgical restoration, through APG, of portal trunk patency (yellow arrows) with adequate portal inflow

**Fig. 4:**
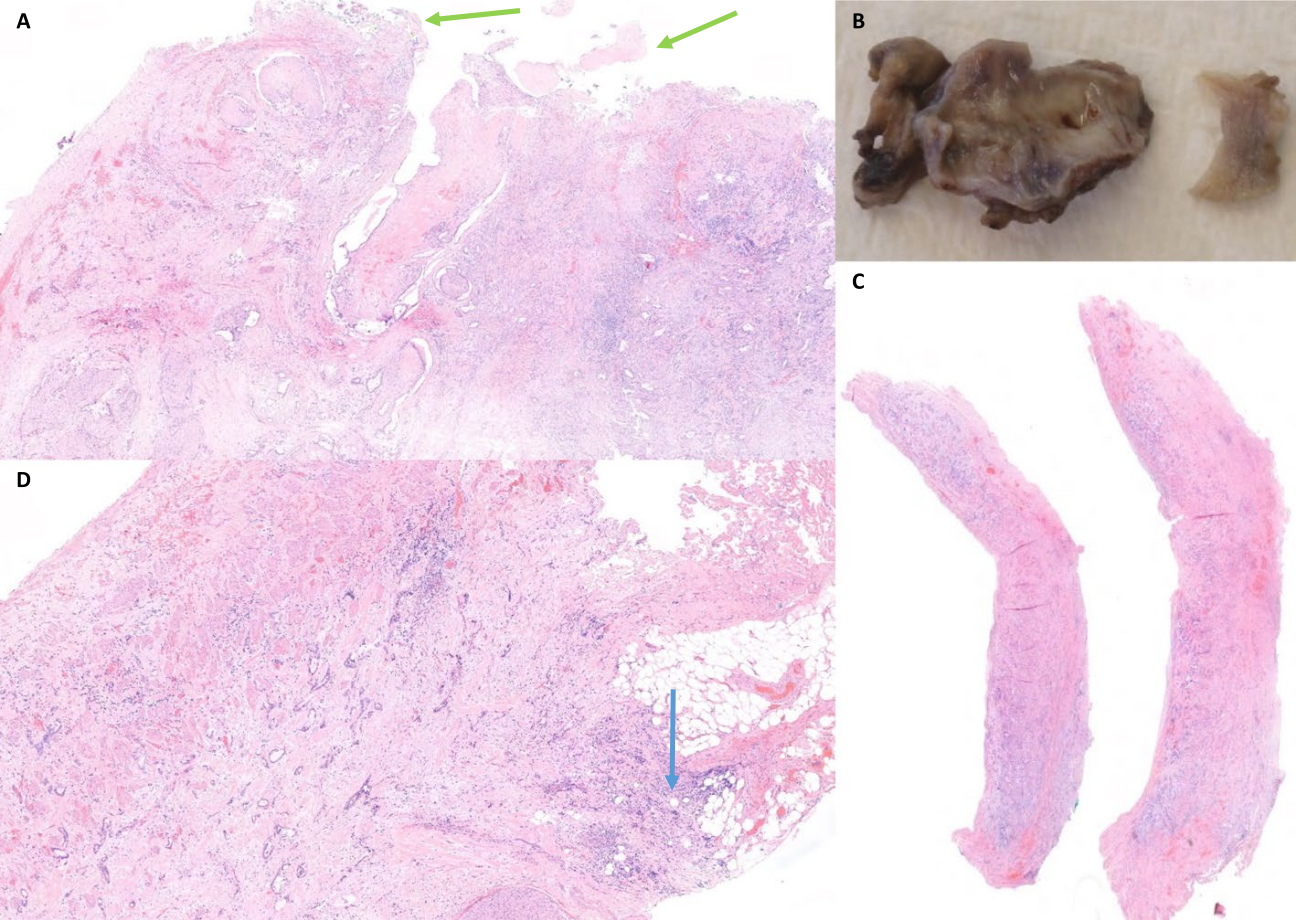
(**A**, **B**, **C**, **D**): Final pathology report. (**A**) Haematoxylin-Eosin staining, 10x. Adenocarcinoma infiltrating the vascular bed (green arrows) on the duodenocephalopancreatectomy specimen (**B**) Portal vein resection (**C**) Haematoxylin-Eosin staining, 4x. Resection of the portal vein with extensive infiltration of adenocarcinoma, serial transversal sections (**D**) Haematoxylin-Eosin staining, 20x. Extensive infiltration of the portal vein and perivascular adipose tissue (blue arrow)

The postoperative course was uneventful. A combination of daily low-dose aspirin (100 mg) and LMWH heparin (1 mg/kg every 24 h) were prescribed since the first postoperative day. After 1 week, contrast CT scan was performed to confirm anastomotic patency (Fig. 3a–c). No clinic signs of portal hypertension or thrombosis were recorded, and the patient was discharged after 16 days. Preoperative and postoperative laboratory values are shown in Table 1. Biliary tract carcinoma (pT3 (L1,V2,On1), pN2 (13/33), G3, R0) resulted at final pathology (Fig. 4 a - d).

**Table 1:** Preoperative and postoperative laboratory values

*Parameters*	*Preoperative*	*Post-APG (0 GPO)*	*Post-APG (1 week)*	*Post-APG (discharge)*
**AST** (5–33 U/L)	25	138	33	28
**ALT** (6–41 U/L)	45	90	22	20
**GGT** (6–40 U/L)	64	156	142	83
**Phosphate alkaline** (33–141 U/L)	160	285	187	145
**Creatinine** (0.5–1.1 mg/dl)	0.73	1.13	0.60	0.62
**PT ratio** (0.9–1.18)	1.20	1.46	1.10	1.18
**APTT ratio** (0.75–1.29)	1.06	0.86	1.07	1.10
**Platelets** (130–400 10^9^/L)	218	254	269	231
**Albumin** (34–48 g/L)	22	20.9	29.4	32.1
**Total bilirubin** (0.1–1 mg/dl)	0.34	0.70	0.94	0.51
**Direct bilirubin** (0.01–0.25 mg/dl)	0.20	0.46	0.43	0.21

## Discussion

Nowadays, the surgical approach to HPB malignancies is dictated by the need to obtain microscopic tumor clearance (R0), which is the most important predictor of favorable oncological outcomes. Several studies showed that an aggressive surgical strategy, including major resections, extended lymphadenectomies, and vascular reconstructions, was associated with improved long-term survival [1]. To achieve a curative R0 resection, technically demanding scenarios such as intraoperative detection of vascular invasion, iatrogenic vascular injuries, and acute vascular thrombosis raised awareness on the theme of vascular reconstruction and vascular substitutes.

A surgical case with extensive portal trunk neoplastic infiltration, rendering both venorrhaphy and end-to-end anastomosis technically unviable, is reported. When simple vascular reconstruction is deemed impossible, an appropriate vascular substitute needs to be promptly available. In case of clear preoperative evidence of vascular invasion, which allows adequate surgical planning, autologous living tissue vascular substitutes (mainly saphenous, renal, and umbilical grafts) are frequently chosen. Nevertheless, great saphenous grafts require additional incisions while renal grafts are reluctantly used due to the potential risk of acute kidney failure. In our case, the great saphenous vein (GSV) could have been harvested with minimal disadvantages but total portal trunk reconstruction with autologous GSV graft would have required additional and extensive graft remodeling [9]. From an anatomical standpoint, the GSV cannot ensure adequate diameter and thickness to effectively interpose a major vessel.

Prosthetic grafts are often chosen as desirable vascular substitutes because of their ready availability, excellent operative maneuverability, and kink resistance. PTFE vascular grafts have been routinely adopted as bypass materials, during cardiovascular procedures, with satisfying results. However, PTFE grafts have been linked to an increased risk of thrombosis and infection often requiring long-term anticoagulation or PTFE removal. Furthermore, several studies described accidental graft gastric penetration and severe abdominal sepsis. PTFE graft infection becomes particularly relevant in patients requiring mesentericoportal axis reconstruction for tumor’s infiltration during pancreaticoduodenctomy. Whipple’s procedure is notoriously linked to high rates of postoperative pancreatic fistula frequently leading to severe intra-abdominal sepsis and hemorrhage. In this scenario, any factor that could increase postoperative infectious morbidity rates should be carefully detected and avoided. These additional risks have restricted PTFE graft use for portal vein reconstruction [4].

Cryopreserved veins (e.g., femoral vein) allografts from deceased donors are a valid alternative option. However, because of frequent shortage of donors and limited availability outside transplant centers, their use is limited [10]. In summary, numerous vascular substitutes are potentially available upon careful evaluation of their inherent disadvantages.

In our case, after an accurate intraoperative assessment of portal vein longitudinal infiltration, we decided to use a parietal peritoneum interposition graft as a backup reconstruction choice. The autologous peritoneal graft (APG) was easily and rapidly harvested from the right diaphragmatic peritoneum, without any additional incision and within the same surgical field. The APG presents several significant advantages. When compared with the abovementioned vascular substitutes, APG is inexpensive, it has a significantly lower risk of graft infection, and it is readily available. This is particularly relevant in case of unplanned vascular resection or in emergency situations. Furthermore, the peritoneum is an extremely compliant tissue that can be effortlessly handled to build tailored grafts with the exact diameter and length required [7]. In recent years, APG graft has been increasingly used by different HPB surgical teams when tackling venous reconstructions. Dokmak et al. [8] proved that parietal peritoneum (PP) grafts are a safe and feasible option when lateral vascular patches are needed. Other series demonstrated favorable surgical outcomes of peritoneal grafts for both IVC lateral reconstruction and replacement. Nevertheless, limited data is available on tubular peritoneum grafts, especially when used as vascular substitutes for mesentericoportal axis reconstruction.

Very few cases of total portal trunk reconstruction using a tubular PP graft have been reported in literature. Dokmak et al. [8, 11] described its use as an interposition graft between the main and the right branch of the portal vein while other case reports applied this technique during emergency situations such as blunt thoracoabdominal trauma causing complete transection of the main portal trunk [12]. Finally, some series reported the use of tubular PP graft in selected cases of mesenterico-portal axis involvement for pancreatic adenocarcinoma.

Nevertheless, the proportion of venous reconstruction is biased in favor of a lateral PP graft reconstruction rather than a tubular PP reconstruction. According to the series presented by Dokmak et al. [8, 11], lateral patch reconstructions presented less postoperative graft’s related complication when compared to tubular patch types. Tubular reconstructions are technically challenging procedures requiring extensive graft remodeling, preparation, and longer operative times when compared with other reconstruction techniques (direct suture, lateral patches, and end-to-end anastomoses). However, when extensive resections are needed, the interposition of a tubular graft is often the only technical strategy that can ensure flow continuity. Tubular interposition PP grafts could play a key role when tackling difficult resections. This holds true for IVC reconstruction in case of malignancies encasing the entire vessel’s circumference. Pulitano et al. [7] highlighted the advantages of APG repair (e.g., lower risk of graft’s thrombosis, ready availability, increased flexibility, and cheaper cost) when compared to other traditional techniques. A literature summary table on the use of autologous peritoneal tubular interposition graft in HPB surgery is reported in Table 2.

**Table 2:** Literature summary table on the use of autologous peritoneal tubular interposition graft for porto-mesenteric reconstruction in HPB surgery

Autologous peritoneal tubular interposition graft: literature review							
**Author, year**	**Cases, number**	**Pathology**	**Surgical procedure**	**Reconstruction site**	**Graft type and harvesting site**	**Mortality**	**Graft occlusion/stenosis**
**Dokmak**, 2015 [8, 11]	3	Malignant disease	Pancreatic and liver resections	MPV	Autologous non-fascial peritoneum, diaphragm	No	2 Occlusion
**Sabuncuoglu**, 2015 [12]	1	Blunt trauma, complete transection of the hepatoduodenal ligament	Ligation of hepatic artery	MPV	Autologous non-fascial peritoneum, right subcostal region	Yes	No
**Zhiying,** 2017 [13]	4	Pancreatic malignancies	Pancreatectomy	PV/SMV	Falciform ligament	No	3 stenosis; 1 occlusion
**Malinka,** 2018 [14]	2	Pancreatic malignancies	Pancreatectomy	PV	Falciform ligament	No	1 patent; 1 occlusion
**De Pauw,** 2021 [15]	1	Pancreatic adenocarcinoma	Pancreaticoduodenenctomy	PV /SMV	Autologous peritoneum, N/A	No	NR

The literature search highlighted APGs’ occlusion and stenosis as the main postoperative complications after successful vascular reconstruction. One of the potential drawbacks associated with APG reconstructions is an increased risk of postoperative short- and long-term thrombosis. Peritoneal grafts are composed of connective tissue coated with a monolayer of the mesothelium. Prolonged blood flow through a mesothelial conduit, which lacks endothelial regulatory properties on hemostasis, could explain the increased risk of APG occlusion. Nevertheless, an accurate balance, between the increased thromboembolic risk and the risk of postoperative bleeding, should be achieved. APG are relatively soft and flexible vascular conduits that could be easily damaged leading to pseudoaneurysm formation and bleeding. In order to exploit APG advantages and minimize potential risk, due to the precarious balance between thrombosis and hemorrhage, careful APG reconstruction, without tension areas, and handling, without tearing the mesothelium, is required. Additional randomized research on postoperative outcomes is needed to further assess potential benefits and limitations among vascular reconstruction techniques.

Overall, APG is a promising surgical innovation that should always be considered in a technically challenging case requiring aggressive surgical resections. This technical report highlights the advantages of APG grafts through which an R0 resection, on both radial and longitudinal vascular margins, was obtained ensuring optimal oncological clearance in absence of postoperative graft-related complications.

## Conclusion

To this day, APG remains a surgical technique not fully exploited despite its potential benefits. The peritoneal patch is easy to access, readily available, and perfectly suited for optimal vascular reconstruction. In our case, APG represented the technical key point that ensured a safe and radical (R0) resection in a complex case of distal cholangiocarcinoma. Peritoneal promising features, as vascular graft, are yet to be fully understood and should be further explored.

## Data Availability

The datasets used and/or analyzed during the current study are available from the corresponding author on reasonable request.

## Abbreviations

HPB: Hepatopancreatobiliary
PTFE: Polytetrafluorethylene
APG: Autologous parietal peritoneum grafts
IVC: Inferior vena cava
PP: Parietal peritoneum
GSV: Great saphenous vein
